# The Effectiveness of Supplementation with Key Vitamins, Minerals, Antioxidants and Specific Nutritional Supplements in COPD—A Review

**DOI:** 10.3390/nu15122741

**Published:** 2023-06-14

**Authors:** Mónika Fekete, Tamás Csípő, Vince Fazekas-Pongor, Ágnes Fehér, Zsófia Szarvas, Csilla Kaposvári, Krisztián Horváth, Andrea Lehoczki, Stefano Tarantini, János Tamás Varga

**Affiliations:** 1 Department of Public Health, Faculty of Medicine, Semmelweis University, 1089 Budapest, Hungary; fekete.monika@med.semmelweis-univ.hu (M.F.); csipo.tamas@med.semmelweis-univ.hu (T.C.); pongor.vince@med.semmelweis-univ.hu (V.F.-P.); trombitasne.feher-agnes@med.semmelweis-univ.hu (Á.F.); szarvas.zsofia_katalin@med.semmelweis-univ.hu (Z.S.); kaposvari.csilla@med.semmelweis-univ.hu (C.K.); horvath.krisztian@med.semmelweis-univ.hu (K.H.); 2 Department of Haematology and Stem Cell Transplantation, National Institute for Haematology and Infectious Diseases, South Pest Central Hospital, 1097 Budapest, Hungary; ceglediandi@freemail.hu; 3 Department of Neurosurgery, The University of Oklahoma Health Sciences Center, Oklahoma City, OK 73104, USA; stefano-tarantini@ouhsc.edu; 4 Department of Health Promotion Sciences, College of Public Health, The University of Oklahoma Health Sciences Center, Oklahoma City, OK 73104, USA; 5 Peggy and Charles Stephenson Oklahoma Cancer Center, Oklahoma City, OK 73104, USA; 6 Department of Pulmonology, Semmelweis University, 1083 Budapest, Hungary

**Keywords:** COPD, vitamins, minerals, antioxidants, omega-3

## Abstract

Currently, an increasing amount of evidence supports the notion that vitamins C, D and E, carotenoids, and omega-3 fatty acids may protect against the progression of chronic respiratory diseases. Although chronic obstructive pulmonary disease (COPD) primarily affects the lung, it is often accompanied by extrapulmonary manifestations such as weight loss and malnutrition, skeletal muscle dysfunction, and an excess of harmful oxidants, which can lead to a decline in quality of life and possible death. Recently, the role of various vitamins, minerals, and antioxidants in mitigating the effects of environmental pollution and smoking has received significant attention. Therefore, this review evaluates the most relevant and up-to-date evidence on this topic. We conducted a literature review between 15 May 2018 and 15 May 2023, using the electronic database PubMed. Our search keywords included COPD, chronic obstructive pulmonary disease, FEV_1_, supplementation: vitamin A, vitamin D, vitamin E, vitamin C, vitamin B, omega-3, minerals, antioxidants, specific nutrient supplementations, clinical trials, and randomized controlled trials (RCTs). We focused on studies that measured the serum levels of vitamins, as these are a more objective measure than patient self-reports. Our findings suggest that the role of appropriate dietary supplements needs to be reconsidered for individuals who are predisposed to or at risk of these conditions.

## 1. Introduction

Chronic obstructive pulmonary disease (COPD) is a preventable and treatable public health issue characterized by progressive bronchial obstruction [1,2]. Increased resistance to airflow is a consequence of a pathologically heightened inflammatory reaction to lung tissue-damaging gases and particles (most commonly from smoking) that are inhaled [2,3]. Acute exacerbations and comorbidities are important prognostic factors for the severity of COPD [2,4]. COPD is an underdiagnosed disease with increasing morbidity and mortality worldwide [4,5], and is projected by the World Health Organization (WHO) to become the third leading cause of death by 2022 [6]. Its causes include changing smoking habits, an ageing population, and certain respiratory infections [7,8]. The burden of COPD, i.e., the number of disability-adjusted life years (DALYs), is significant in society, patients have a significantly lower quality of life and decreased work capacity compared to non-COPD patients of the same age [9,10,11].

COPD is not only a disease characterized by chronic, progressive loss of respiratory function and respiratory and systemic inflammation; it can also affect the entire body, leading to weight loss in approximately 20–50% of cases, which can become pathological [3,12]. Correcting malnutrition is an important part of maintenance therapy for COPD, and has been shown to improve exercise tolerance, quality of life, and survival in patients [6,13,14,15]. Numerous studies have revealed the benefits of a diet rich in antioxidants in COPD, including vitamins A, C, and E, beta-carotene, and micronutrients such as magnesium, selenium, and zinc [14,16,17,18]. In addition, the quality of the diet and the intake of long-chain polyunsaturated fatty acids (PUFAs; omega-3), which can reduce inflammation in many ways, are very important factors for chronically ill patients [19,20]. Acute exacerbations in COPD patients are primarily caused by various bacterial and viral infections, and vitamin D deficiency can increase the risk of infections as well as chronic inflammation [21], which is extremely dangerous for severely ill COPD patients with low forced expiratory volume in one-second (FEV_1_) values [22,23]. Sarcopenia, weight loss, low body mass index (BMI), and low fat-free mass (FFM) justify the need for early complex dietary interventions, oral nutritional supplements, and supplementation with specific nutrients such as vitamins, antioxidants, minerals, etc. [24].

The quality of life of COPD patients is influenced by a number of factors, such as the nutritional status of patients, the quality (e.g., energy, protein, vitamin, and antioxidant content) and quantity of their food intake, and their level of physical activity, as these are all modifiable risk factors for systemic inflammation that can contribute to reducing inflammation during COPD progression [13,25,26]. Over the past two decades, several studies have reported that the risk of COPD is associated with antioxidant-rich vitamins and antioxidant-rich diets [13,27,28,29,30]. Low vitamin intake has been reported to reduce the effectiveness of the immune system and increase the number of exacerbations [31]. In addition, higher vegetable and fruit intake is associated with improved respiratory function values and lower mortality in COPD patients [32]. The effectiveness of supplementation with the most important vitamins, minerals, antioxidants, and specific nutritional supplements in COPD is not in question, as it is associated with respiratory symptoms, exacerbation frequency, lung function, and quality of life [32,33]. The aim of this literature review is to update our knowledge on the relationship between the most important vitamins, minerals, antioxidants, and specific nutritional supplements and COPD, and to explore their potential role in the pathomechanism of COPD.

## 2. Methods

We conducted a literature review between 15 May 2018 and 15 May 2023 using the electronic database PubMed. Our search keywords included COPD, chronic obstructive pulmonary disease, FEV_1_, supplementation: vitamin A, vitamin D, vitamin E, vitamin C, vitamin B, omega-3, minerals, antioxidants, specific nutrient supplementations, clinical trials, and randomized controlled trials (RCTs). We focused on studies that measured serum levels of vitamins, as these are a more objective measure than patient self-reports. Other nutritional factors, such as fish consumption, calorie supplementation, or protein replacement, which are also associated with the pathomechanism and outcomes of COPD, were not included in this review. In addition, in vivo and animal studies are not part of this review.

## 3. Results

### 3.1. Vitamin A

No human RCTs or clinical trials on the association between COPD and vitamin A supplementation were found in the PubMed database in the past five years. However, one article discussed the very important anti-inflammatory role of this antioxidant vitamin, as there has been growing interest in the role of antioxidant vitamins in respiratory health. The National Health and Nutrition Examination Survey (NHANES) III and the ongoing NHANES data comprehensive analysis is the largest study in this area, including data from more than 34,000 participants; it examines whether serum antioxidant levels of vitamins A, C, D, and E are associated with respiratory morbidity and mortality in adults in the United States [34]. Serum concentrations of vitamins A, C, and E were measured using isocratic high-performance liquid chromatography. They examined whether lower serum levels of vitamins A, C, D, and E were associated with respiratory illness and mortality. For vitamin A, although serum antioxidant vitamin levels were significantly higher among those who took vitamin A supplements than in those who did not, there was no clear statistical evidence that vitamin A supplementation was necessary for the beneficial effects of vitamin A on the lung. In general, lower serum levels of vitamins A, C, D, and E are associated with increased respiratory morbidity and/or mortality in US adults. In addition, influenza/pneumonia-related mortality increased with decreasing serum levels of vitamin A in all participants (total adjusted hazard ratio [aHR]: 1.21, 95% CI: 0.99–1.48) [34].

### 3.2. Vitamin B

A cross-sectional study of 1201 COPD patients examined the association between vitamin B intake and frailty [35]. The results showed that COPD patients with lower vitamin B_6_ intake had a higher risk of frailty (aOR = 0.80, 95% CI = 0.66–0.95, *p* = 0.013). However, intakes of vitamins B_1_, B_2_, niacin, total folic acid, and vitamin B_12_ were not associated with changes in frailty in COPD patients [35]. Another study investigated the effect of vitamin B_12_ supplementation on exercise and natriuretic peptide levels after an 8-week intervention. The results showed that vitamin B_12_ supplementation significantly altered the temporal secretion of NT-proBNP (natriuretic peptide) during treatment (*p* = 0.048), suggesting that vitamin B_12_ supplementation should be considered for COPD patients due to the increased risk of cardiovascular morbidity [36].

### 3.3. Vitamin C

Researchers examined the role of long-term high-dose vitamin C (2 g/day, 500 mg four times daily) in patients with COPD (*n* = 26), during a 6-month follow-up and an interim follow-up every 3 months. They found that the number of exacerbations was lower in patients who received high-dose and regular vitamin C, with an odds ratio (OR) of 5.26 [2.44–11.31] and a *p* < 0.0001 [37]. In another study [38], based on an estimate of total vitamin C consumption, it was found that patients with chronic respiratory disease consumed significantly less vitamin C than healthy controls. A comparison of the results of the two groups showed that the serum vitamin C concentrations in patients with chronic respiratory disease were significantly lower than those of the control group, and the blood oxygen saturation in patients with chronic respiratory disease was significantly lower. Furthermore, patients with chronic respiratory disease had significantly higher levels of C-reactive protein (CRP) and other inflammatory markers, which are risk factors for predicting metabolic complications [38].

In another study [39], a multi-component nutritional support containing vitamin C was administered to patients with moderate-to-severe COPD. This nutritional intervention improved skeletal muscle mass and strength, increased the quality of life of patients, and reduced blood levels of inflammatory cytokines (CRP, IL-6, TNF-alpha) without exercise. A whey protein drink containing 275 mg of elemental magnesium, 685 mg of vitamin C, and 15.9 g of whey protein was given to the intervention group for 8 weeks [39]. In another study [40], researchers supplemented COPD patients with Nigella sativa (black seed oil), orally, twice daily (1 g of 100% pure cold-pressed black seed oil), in addition to standard COPD medications. The remarkably potent biological activity of Nigella sativa is due to its bioactive compounds, namely selenium, vitamin E, retinol, vitamin C, and carotenoids, which have antioxidant, anti-inflammatory, and anticancer properties. Its effects are diverse, including antispasmodic, bronchodilator, immunomodulatory, antidiabetic, antihistamine, antimicrobial, anti-inflammatory, and antioxidant effects. The aim of the study was to investigate the effects of this extract on respiratory function, airway inflammation, and the oxidant–antioxidant balance as a complementary therapy. The results of the study showed several positive effects in COPD, particularly a significant improvement in lung function and airway inflammation. Significant reductions in interleukin-6 (IL-6) and tumor necrosis factor-alpha (TNF-alpha) were observed, as well as a significant increase in antioxidants; superoxide dismutase (SOD), catalase (CAT), reduced glutathione (GSH), glutathione peroxidase (GPx), vitamins C and E, and pulmonary function test (PFT) values were also significantly improved compared to the control group and baseline levels. Black seed oil was generally well tolerated by patients, and none of the participants reported any side effects [40].

Researchers examined the effect of intravenous vitamin C administration on exercise-induced redox balance, inflammation, exertional dyspnea, neuromuscular fatigue, and exercise tolerance in patients with COPD [41]. Eight patients performed constant load cycling (∼80% peak power output) after intravenous administration of vitamin C (2 g) or saline (placebo) infusion. Vitamin C increased superoxide dismutase (a marker of endogenous antioxidant capacity) by 129%, and moderated the plasma concentration of CRP during exercise, but did not affect exercise-induced increases in lipid peroxidation (malondialdehyde) or free radicals. Therefore, although vitamin C reduced inflammatory markers and improved resistance to neuromuscular fatigue, it did not improve exercise-induced dyspnea and cycling exercise tolerance in patients with COPD. Vitamin C prevented the exercise-induced increase in C-reactive protein, and reduced the development of fatigue, which was a critical determinant of exercise tolerance (Table 1).

### 3.4. Vitamin D

Vitamin D has anti-inflammatory, immunomodulatory, and antimicrobial effects. Vitamin D deficiency occurs in a significant number of patients with COPD, making it particularly important to measure and evaluate vitamin D levels in chronic respiratory patients. A recent RCT study found that vitamin D supplementation did not reduce the number of exacerbations in COPD or affect physical performance in COPD patients with vitamin D deficiency [42]. However, another study found evidence of the likely benefits of vitamin D supplementation in patients with severe vitamin D deficiency [43]. A recent RCT study also reported clear and proven benefits of vitamin D supplementation in a group of COPD patients, with significant differences in the quality of life after 2 months (*p* < 0.001) and 6 months (*p* < 0.001) in patients who consumed 50,000 IU of vitamin D per week for 8 weeks and then received monthly vitamin D supplementation [44]. It is known that suboptimal vitamin D status (25(OH)D ≤ 50 nmol/L) is common among COPD patients, but the association between vitamin D levels and COPD exacerbations continues to produce conflicting results. Therefore, a study was conducted to assess the correlation between vitamin D levels and respiratory symptoms, exacerbations, and imaging features [45]. Their results showed that vitamin D deficiency was significantly associated with poorer quality of life, increased dyspnea, reduced exercise tolerance, and increased frequency of severe exacerbations. In addition, vitamin D deficiency was associated with the thickening of the airway walls on chest CT scans, which may affect the role of vitamin D levels as a risk factor for COPD exacerbations [45]. Another RCT study hypothesized that vitamin D supplementation reduced elastin degradation, especially in COPD patients with vitamin D deficiency, which was evaluated by measuring plasma desmosine levels. However, their hypothesis was contradicted, as the results showed no significant effect of vitamin D supplementation on elastin degradation compared to the placebo group [46]. Another study sought to determine how high-dose vitamin D (300,000 IU) supplementation affected systemic inflammatory biomarkers (IL-6, IL-8, CRP) in patients with chronic obstructive pulmonary disease experiencing acute exacerbation [47]. Their results showed a significant reduction in IL-6 levels in the vitamin D group on day 6 compared to the placebo group (*p* = 0.02), but no significant difference was observed in IL-8 and hs-CRP, mMRC scale, length of hospital stay, and mortality rate [47]. An RCT study aimed to investigate whether or not vitamin D supplementation enhanced the effects of endurance training in COPD patients. Its results showed that vitamin D3 supplementation did not affect any of the measured outcome parameters related to the muscle response to endurance training in COPD patients [48] (Table 2).

### 3.5. Antioxidant Supplementation in COPD

Coenzyme Q10 (CoQ10) can potentially be used as an antioxidant and anti-inflammatory agent in the treatment of diseases in which oxidative stress is present. Very few clinical trials have investigated CoQ10 supplementation, but De Benedetto et al. demonstrated in an RCT study that a combination of CoQ10 and creatine for two months significantly improved body composition, dyspnea, physical performance, daily activities, and showed positive changes in plasma metabolic profiles in COPD patients receiving long-term oxygen therapy [49]. Resveratrol is another important antioxidant that can improve metabolic health in patients by enhancing mitochondrial function in muscles. Therefore, researchers examined the effects of four weeks of resveratrol supplementation in COPD patients [50]. Their results showed that plasma C-reactive protein and kynurenine did not change after resveratrol supplementation, while markers of glycolysis and lipolysis increased significantly. Body weight decreased due to a decrease in lean body mass. Their results did not confirm the previously reported positive effects of resveratrol on skeletal muscle mitochondrial function in COPD patients [50]. Crocin from saffron (*Crocus sativus* L.) is a herbal supplement with antioxidant and anti-inflammatory properties that appears to be effective in improving oxidant/antioxidant balance and systemic inflammation in COPD patients. After a 12-week intervention, crocin reduced the total oxidant status and nuclear factor kappa B (NF-κB) serum levels and increased the total antioxidant capacity and exercise capacity (6MWD) of COPD patients [51]. Fares Gouzi et al. administered various antioxidant supplements, including vitamins C and E, zinc, and selenium, to COPD patients during pulmonary rehabilitation. The patients showed significant improvements in muscle strength, and the antioxidant cocktail improved muscle function and reduced the prevalence of muscle weakness [52]. In another study, 12 weeks of oral beta-alanine supplementation effectively increased muscle carnosine (an endogenous antioxidant), but did not result in beneficial changes in physical performance, quadriceps function, or oxidative/carbonyl stress in muscle [53]. Oligomer proanthocyanidins (OPCs) extracted from grape seeds have high antioxidant capacity, so Meng Chun Lu et al. evaluated their effects in COPD. Patients were given 150 mg/day of OPCs orally for 8 weeks, which significantly reduced malondialdehyde, superoxide dismutase concentration, and the total cholesterol (TC)/high-density lipoprotein cholesterol (HDL-C) ratio. Overall, the treatment increased the antioxidant capacity and improved the lipid profile of COPD patients [54]. In another study, black seed oil supplementation resulted in significant reductions in oxidant and inflammatory parameters in COPD patients, and their results suggest that black seed oil supplementation may be an effective adjunctive therapy in COPD [40]. Quercetin is a plant flavonoid that has strong antioxidant and anti-inflammatory properties. Therefore, MK Han et al. administered quercetin to COPD patients at doses of 500, 1000, 2000 mg/day for 1 week. Blood tests showed that quercetin up to 2000 mg/day was safely tolerated, but the study had the disadvantage of a small sample size and a relatively short duration of administration, with only one week of this supplementation in patients [55]. Aslani MR et al. evaluated a herbal supplement with anti-inflammatory and antioxidant properties (*Crocus sativus* L. crocin supplement) in COPD patients receiving 15 mg twice daily for 12 weeks. Crocin significantly improved the respiratory function tests, exercise tolerance of patients (6MWD) and serum levels of IL-6 compared to the placebo group, and significantly reduced the patients’ serum TNF-α levels compared to the placebo group. Their results suggest that crocin supplementation improves exercise capacity and respiratory function in COPD patients with reduced serum levels of inflammatory factors [56] (Table 3).

### 3.6. Polyunsaturated Fatty Acids (PUFAs)

PUFAs have demonstrated multifaceted properties. Firstly, they have anti-inflammatory effects [57]. Secondly, they have been found to have bronchodilator properties [20,57]. Thirdly, they mitigate hypoxia-induced pulmonary vasoconstriction through the alteration of cellular membrane composition, and their supplementation has been shown to have beneficial effects in various diseases [20]. Kim J. S. et al. postulated that PUFA supplementation enhanced systemic endothelial function in patients with chronic obstructive pulmonary disease, given the well-established pathogenic role of endothelial dysfunction in COPD. Nonetheless, the impact of PUFA supplementation on endothelial function in this specific patient cohort remains unexplored [58]. COPD patients were administered high-dose fish oil capsules daily for six months; however, this intervention failed to elicit significant modifications in systemic endothelial function in COPD, per the investigation. Nonetheless, the existence of biological and clinical effects associated with PUFA supplementation was confirmed, as shown by a significant improvement in the quality of life of patients (SGRQ) and an increase in the number of plasma CD31+ endothelial microparticles (EMPs) [58]. Engelen M et al. [59] also used a randomized controlled trial to determine whether high-dose (3.5 g/day) PUFA supplementation improved protein homeostasis and positively modulated protein metabolism in individuals with COPD. Their findings indicated that patients tolerated the high-dose omega-3 supplementation well, inducing favorable changes in protein homeostasis within the COPD patient group in a partly dose-dependent manner. Notably, limb lean mass increased, although muscle function did not demonstrate significant alterations [59]. In a cross-sectional study, Fekete et al. used a self-developed questionnaire to investigate the impact of self-reported omega-3 supplementation over the past 6 months among COPD patients. They observed correlations between regular low-dose PUFA supplementation (0.5 g/day) and improved quality of life, nutritional status, inflammatory parameters, lipid profiles, as well as the use of inhaled medication [20]. In an RCT study, Ogasawara T et al. [60] aimed to investigate the effects of oral supplementation with 1 g of Eicosapentaenoic acid (EPA)-enriched oral nutritional supplements (ONS) daily, during hospitalization, on lean body mass index (LBMI) and skeletal muscle mass index (SMI) compared to a control group. Their findings indicated that EPA-enriched ONS did not confer significant benefits in preserving lean body mass (LBM) and muscle mass during short-term hospitalization in patients with exacerbation, compared to EPA-free ONS. However, longer-term supplementation with EPA may play a crucial role in restoring skeletal muscle mass following COPD exacerbation, as changes in skeletal muscle mass index (SMI) demonstrated a significant correlation with length of hospital stay in the EPA group; however, no such correlation was observed in the control group (r = 0.53, *p* = 0.008, and r = −0.09, *p* = 0.70 [60] (Table 4).

### 3.7. Magnesium and Sodium Nitrate

Magnesium deficiency is known to affect quality of life, inflammation levels, degree of obstruction, exacerbation frequency, and physical performance in patients with COPD [61]. Zanforlini BM et al. aimed to answer whether oral supplementation with 300 mg/day magnesium citrate affected respiratory function, physical performance, and quality of life. Their results showed that magnesium supplementation may have a potential anti-inflammatory role in COPD, but it did not affect the quality of life of patients, and no significant changes were found in respiratory parameters [61]. Beijers RJHCG et al. [62] investigated the effect of 1-week sodium nitrate supplementation on physical performance and cardiac biomarkers in a group of COPD patients at increased cardiometabolic risk (abdominal obesity prevalence: 83.3%). Their results showed no significant favorable effect on blood pressure, cardiac biomarkers, or exercise tolerance in patients with mild to moderate COPD during the study period [62] (Table 5).

## 4. Discussion

There is growing evidence to support the notion that various vitamins, minerals, antioxidants such as vitamins C, D and E, carotenoids, etc., and specific nutrient supplements such as omega-3 fatty acids may protect against the progression of chronic respiratory diseases. Oxidative stress plays an important role in the development of chronic diseases [63], and evidence suggests that improper nutrition, including malnutrition and obesity, can increase levels of oxidative stress, systemic inflammation, and the risk of chronic diseases [63]. Oxidative stress, which results from an imbalance between reactive oxygen species (ROS; Table 6) and antioxidants, can lead to tissue damage, airway inflammation, exacerbation of COPD, and pathological immune responses [64,65]. If antioxidants are unable to eliminate endogenous and exogenous ROS molecules, pathological levels of oxidative stress develop, which increases the risk of exacerbations in COPD. ROS reaction with various biomolecules such as proteins, lipids, and DNA may cause cell injury, leading to apoptosis and necrosis [28,65,66]. Serum concentrations of antioxidants have been shown to correlate positively with FEV_1_ in patients with COPD, and supplementation with antioxidants such as vitamin C improves the symptoms of the disease [64,67,68].

A number of pathogenic processes are known to play a role in the development and progression of COPD, including imbalances in the antioxidant capacity of harmful oxidants, inflammation, protease/antiprotease imbalance, transformation of immune responses, and emphysematous destruction of lung parenchyma [4,69,70]. Although COPD primarily affects the lung, it is often accompanied by extrapulmonary manifestations such as weight loss and malnutrition, skeletal muscle dysfunction, which can lead to declining quality of life and eventual mortality [71,72,73]. In addition, other chronic diseases (known as comorbidities), including cardiovascular diseases, especially coronary artery disease, osteoporosis, metabolic syndrome, depression, and lung cancer, are also highly prevalent in COPD patients and act to worsen disease outcome [8,74]. The underlying pathogenesis of these diseases is characterized by low-grade systemic inflammation, which plays a crucial role in disease prognosis and ultimate outcome [1,26,74,75,76,77].

It is noteworthy that active smokers are more likely to follow unhealthy dietary habits than those who have quit smoking [78] and have higher levels of oxidative stress, which can be reduced by changing their diet and supplementing it with vitamins and antioxidants [3]. Among active smokers, higher levels of oxidative markers are correlated with poorer lung function [30], whereas higher serum levels of antioxidant enzymes (such as catalase, superoxide dismutase, and glutathione peroxidase) are positively associated with lung function [79]. In addition, poor diet quality and nutrient deficiencies in chronic respiratory patients may be related to disease-specific factors such as increased symptoms (e.g., shortness of breath, fatigue, anxiety, depression, loss of appetite, impaired taste, dysphagia, and increased physical inactivity), which can be improved via dietary interventions, vitamin and antioxidant supplementation, and specific dietary supplements, and subsequently may result in positive changes in disease pathogenesis [71].

Several studies have examined the role of antioxidant vitamins in the pathogenesis of respiratory diseases, but the findings are conflicting [80,81]. Many of these studies relied on self-reported dietary intake of antioxidant vitamins rather than objectively measured serum vitamin levels. Vitamins A, C, and E are the main non-enzymatic antioxidants, but vitamin D (calciferol) has also been shown to have antioxidant properties; thus, all four vitamins are antioxidant in nature [82]. The primary source of vitamin A, C, and E precursors is the diet, namely fresh vegetables and fruits [83]. In addition to neutralizing free radicals, vitamin A and α-tocopherol also reduce lipid peroxidation, and vitamin A plays an important role in the proliferation of lung epithelial cells [84]. Evidence from animal models suggests a possible link between vitamin A and COPD [85], but epidemiological studies examining the relationship between vitamin A and respiratory disorders in adults have reported conflicting results [34]. In an NHANES study, after adjusting for confounding factors, dietary consumption of the recommended amount of vitamin A reduced the risk of developing emphysema; supplementation with the appropriate amount of vitamin A had an anti-inflammatory effect [86], but there was no clear statistical evidence that artificial supplementation with vitamin A was necessary for the beneficial effects of vitamin A in individuals with respiratory disease [34]. Antioxidant vitamins and certain trace elements such as iron, zinc, copper, and selenium synergistically support the defensive activity of immune cells, and reduced levels of these micronutrients in the serum can negatively affect the inflammatory cascade and lung health [87].

Vitamin C has long been recognized as an antioxidant in COPD, and we know that the main drugs for the disease, such as antibiotics, corticosteroids, and bronchodilators, do not act against oxidative stress [88]. Vitamin C supplementation improves respiratory function and the oxidant/antioxidant balance, which is an important prognostic factor in COPD [88]. Several factors, including smoking and air pollution, have been shown to increase systemic oxidative stress levels in patients, but antioxidant supplementation can improve COPD symptoms [28]. The results of meta-analyses have shown that vitamin C supplementation is significantly and positively correlated with improved lung function in patients with COPD, with more than 400 mg vitamin C supplementation daily significantly improving lung function; meanwhile, patients supplemented with less than 400 mg of vitamin C did not experience significant changes [88]. Evidence supports that vitamin C supplementation has clinically significant benefits in the treatment of COPD [89]. Oxidative stress is widespread in COPD, exacerbated by hypoxia and infection, and plays a crucial role in lung tissue damage [90]. Serum levels of the major antioxidants (vitamins C and E) are lower in smokers than in non-smokers [91]. In addition, vitamin C has shown a synergistic effect with vitamin E in antioxidant activity [92]. Vitamin C supplementation can improve serum antioxidant capacity and quality of life in COPD patients, and reduce COPD mortality rates.

Approximately 20–50% of patients with COPD suffer from weight loss, as well as protein and calorie deficiency [93], which contributes to respiratory muscle dysfunction, disease severity, disability progression, and ultimately, disease mortality [14]. Antioxidant imbalances can lead to systemic inflammation, which has been shown to damage muscles, reduce respiratory capacity, and reduce lung function, ultimately leading to muscle dysfunction and atrophy [94]. Undernutrition exacerbates disease progression and increases oxidative stress in patients, also increasing disease severity. Supplementation with vitamin C can improve their nutritional status. In a US study with more than 34,000 participants, lower serum vitamin C levels increased the risk of exacerbations (adjusted odds ratio (aOR): 1.08, 95% CI: 1.01–1.16), and lower serum vitamin C levels were associated with increased mortality from chronic lower respiratory diseases (adjusted relative hazard (aHR): 1.27, 95% CI: 1.07–1.51). In a combined analysis, vitamin C and vitamin D deficiency were associated with influenza/pneumonia-related mortality, which doubled the risk of death [34].

Several factors, including smoking and air pollution, have been shown to increase systemic oxidative stress in patients with COPD, and antioxidant supplementation can improve COPD symptoms. Non-enzymatic antioxidants include vitamins C and E, carotene, and glutathione, which are present at lower levels in smokers than in non-smokers [95]. Vitamin C has a synergistic effect with vitamin E on antioxidant activity, and elevated serum levels of vitamin E show protection against COPD mortality [96]. After an analysis of NHANES III data, lower serum levels of alpha-tocopherol were associated with an increase in exacerbations and chronic bronchitis/emphysema, although the effect of alpha-tocopherol was observed and described as anti-inflammatory primarily in smokers [34]. Vitamin E intake is positively correlated with higher FEV_1_, alleviates coughing, and long-term vitamin E supplementation reduces oxidative stress markers in the urine of male smokers [97]. In a large cross-sectional study in the United States, higher intake of vitamin E showed an independent association with a reduction in COPD prevalence, and the study also revealed that the average daily total vitamin E intake in the studied population was 8.66 mg, well below the recommended 15 mg/day [98].

The role of vitamin D clearly arises in the pathogenesis and clinical course of COPD, as there is evidence that vitamin D supplementation may play a potential role in preventing COPD exacerbations [99]. Most studies suggest that vitamin D deficiency appears and is more severe in COPD patients compared to healthy controls [22,99,100]. Research suggests the importance of regular monitoring of vitamin D status in COPD [99,100,101]. Although a number of drugs such as theophylline, long-acting beta2-agonists (LABA), and inhaled corticosteroids (ICS) effectively treat COPD [102], vitamin D is currently also considered to be a kind of drug with systemic and antioxidant effects [99,103,104]. Levels of 25(OH)D are associated with respiratory functional parameters (FEV_1_; FEV_1_/FVC), exercise tolerance (6MWD), and quality of life in COPD [45]. Vitamin D deficiency can significantly affect quality of life; therefore, vitamin D supplementation is of paramount importance in this patient group [105]. Several studies report that suboptimal vitamin D status is also associated with several respiratory diseases, including viral respiratory infections, lung cancer, and asthma, as vitamin D plays a significant role in the respiratory system, wherein it can influence lung cell function and immune response [82,106,107]. High local concentrations of vitamin 25(OH)D in the lung may play a role in the regulation of cell proliferation and differentiation, the production of antimicrobial substances, and the regulation of proinflammatory/inflammatory cytokine production [108].

Vitamin D has been shown to reduce the level of oxidative stress and the number of circulating interleukins (e.g., IL-5,6,9,13), which means that vitamin D treatment of COPD patients can improve their symptoms [101]. Khan et al. revealed a relationship between serum vitamin D levels and COPD progression [109]. The results from this study showed that prolonged vitamin D supplementation in patients with COPD could reduce the frequency of acute exacerbations and improve respiratory function, but did not alleviate dyspnea or the volume and color of sputum production [109]. A meta-analysis by Xiaoyan Li et al. found that vitamin D could improve respiratory function, 6 min walk distance, and quality of life (CAT scores), and reduce the number of acute exacerbations and amount of sputum production [110]. In addition, the main function of the medications most commonly used in the therapy of COPD patients (ICS, LABA, theophylline) is to reduce inflammation and relax bronchioles, ultimately reducing airway resistance. Vitamin D can increase the serum concentration of these medications, thereby improving respiratory function, symptoms, and quality of life [110].

In COPD, it is particularly important to address muscle weakness, which is associated with an increased mortality risk. Vitamin D can affect muscle function [111], and its supplementation can have a beneficial effect on increasing muscle strength and improving oxygen utilization [112]. Hypovitaminosis D induces skeletal muscle weakness (COPD patients are already physically inactive), and with advancing age, skeletal muscle vitamin D receptor expression decreases, which activates calcium (Ca) channels and improves muscle contraction by influencing intracellular Ca levels in normal vitamin D status [113]. Additionally, vitamin D suppresses the production of matrix metalloproteinase-9 (MMP9) in keratinocytes in response to tumor necrosis factor-alpha (TNF-α), which if not checked can lead to damage to the lung parenchyma [114]. It is worth noting that low serum 25(OH)D levels occur in up to two-thirds (60–75%) of severe COPD patients [115], so vitamin D deficiency is frequently observed in COPD patients who are already in a serious condition [100]. Furthermore, this patient group is generally characterized by a high number of comorbidities [73], which can significantly influence the severity of COPD. Chronic diseases such as cardiovascular diseases, osteoporosis, and diabetes are also associated with vitamin D deficiency [116,117]. Meta-analyses have shown that maintaining normal levels of vitamin D can reduce the risk of developing respiratory infections in COPD, improve respiratory function, and decrease the frequency of acute exacerbations, ultimately improving the quality of life of COPD patients and playing an essential role in the proper functioning of the immune system [101,110,118].

Polyunsaturated fatty acids (PUFAs; omega-3 oils) are important components of the Mediterranean diet, which has long been considered the healthiest diet and has a number of health benefits, largely due to its vitamin, antioxidant, protein, fiber, and moderate fat content, mostly consisting of monounsaturated and omega-3 polyunsaturated fatty acids [29]. The Mediterranean diet also protects against the harmful effects of smoking, both for active smokers and for those exposed to passive smoking [119]. Increasing one’s intake of omega-3 polyunsaturated fatty acids reduces inflammation, modulates fatty acid homeostasis in cell membranes, modifies eicosanoid metabolism pathways, and ultimately reduces the severity of clinical symptoms in chronic respiratory diseases [20,120]. PUFAs have received considerable attention for their anti-inflammatory properties and their ability to prevent blood clotting, thereby reducing the risk of cardiovascular diseases (which are common comorbidities in COPD) [121]. They are essential from a nutritional point of view and are obtained from external sources, mainly from seafood (e.g., fatty fish and plant seeds) or through supplementation [122]. Because of their anti-inflammatory properties, they are clinically useful for the treatment of various chronic inflammatory diseases in clinical settings, including cardiovascular diseases, asthma, rheumatoid arthritis, and inflammatory bowel diseases such as Crohn’s disease [20]. Conversely, the pro-inflammatory effects of omega-6 fatty acids, including linoleic acid and its long-chain derivative arachidonic acid, which are mainly found in vegetable oils (e.g., soybean, corn, and sunflower oil), dairy products, and eggs [123], have been described. It is believed that the Western diet, with its increased consumption of omega-6 fatty acids and decreased consumption of omega-3 fatty acids, has contributed to the global increase in chronic inflammatory diseases [124].

In stable COPD, higher levels of circulating inflammatory parameters (such as IL-6, CRP) were associated with higher omega-6 intake, whereas lower serum levels of TNF-α were significantly correlated with omega-3 PUFA intake [125]. In an RCT involving 86 COPD patients, one group received omega-3, vitamin D, and leucine supplementation combined with high-intensity exercise for four months, while the other group did not receive dietary supplementation. Their results after four months were significant in terms of exercise tolerance, weight gain, serum vitamin D levels, and eicosapentaenoic acid and docosahexaenoic acid levels [126]. Conversely, the high saturated fat intake characteristic of a Western-type diet may exacerbate airway inflammation in chronic respiratory patients [127,128].

## 5. Limitations of the Study

Due to manuscript length, limitations conference abstracts and meta-analyses, systematic reviews were not included in this review. There is a lot of variation in the research descriptions of the study programs, as well as what is measured afterwards and when, thus an exact comparison of studies is not possible. There is probably a selection bias because of the different physical condition of the patients.

## 6. Conclusions

COPD is a chronic and progressively obstructive disease characterized by inflammation and flow limitation of the small airways, accompanied by systemic inflammation and the presence of multiple chronic comorbidities. It appears that smoking cessation is not a sufficient intervention in the progression of the inflammatory process in COPD, which is induced by oxidative stress and constitutes the most important basis of COPD pathophysiology. Antioxidant therapy and/or a diet rich in intensive antioxidant elements may affect the inflammatory processes and the progression of COPD. Several foods and their constituents, such as vitamins, antioxidants, and specific dietary supplements (with anti-inflammatory, antioxidant, and beneficial metabolic properties that are characteristic of the Mediterranean diet), have been associated with improved respiratory function in numerous studies, including those involving COPD patients. These have been shown to be important in both prevention and treatment. Additionally, they have beneficial effects against systemic inflammation, oxidative stress, mitochondrial dysfunction, and potential immune system support, thus making them a potential option for the treatment of some COPD patients. Overall, our review’s results demonstrate that supplementation with various vitamins, antioxidants, and specific dietary supplements has a positive effect on COPD symptoms, respiratory function, exacerbation, and quality of life. Increased vitamin intake may also reduce the annual decline in FEV_1_. Although the underlying mechanisms of these effects are not yet fully understood, these results may provide a basis for the development of drugs to modify or prevent COPD. Therefore, high vitamin intake and dietary interventions in pursuit of high vitamin intake may present alternative approaches to COPD management.

## Figures and Tables

**Table 1:** Studies on vitamin C supplementation in COPD.

Study	Design	Mean Follow-Up	Country	Sample Size	Average Age (Year)	Sex Male/ Female	Intervention	Main Results
Abuhajar SM et al. [38]	case–control study	-	Gaza Strip, Palestine	52 + 52	44.17 ± 12.98; 43.40 ± 12.39	53.8%/46.2%	-	CRD patients had significantly lower plasma concentrations of vitamins C and CRP than controls (18.43 ± 11.93 μgm/mL vs. 24.06 ± 11.19 μgm/mL, *p* = 0.025); CRP (5.98 ± 8.84 mg/L vs. 1.87 ± 1.96 mg/L, *p* = 0.001).
Ahmadi A et al. [39]	single-blind, randomized trial study	8 weeks	Shiraz, Iran	23 + 23	63.47 ± 7.24; 62.08 ± 7.0	100% male	Received 250 mL of whey beverage fortified with magnesium and vitamin C, daily	IL-6 levels were significantly reduced in the intervention group compared to the control group, and an improvement in health-related quality of life was observed in the intervention group.
Al-Azzawi MA et al. [40]	randomized, controlled, double-blind clinical trial	3 months	Egypt	44 + 47	53.74 ± 4.68; 55.18 ± 4.27	69.2%/ 30.8%	Oral administration of 1 g of 100% pure, cold-pressed black seed oil twice daily in addition to standard COPD medication	Significant reduction in oxidant and inflammatory markers; TBARS, PC content, IL-6, TNF-α a significant increase in antioxidants; SOD, CAT, GSH, GPx vitamins C and E, and a significant improvement in PFTs versus control group and baseline levels.
Hureau TJ et al. [41]	cross-sectional study	no follow-up	Utah, United States	8	65 ± 3	no data available	Intravenous VitC (2 g) during cycling exercise	VitC increased superoxide dismutase by 129% and mitigated CRP in the plasma during exercise, but failed to alter the exercise-induced increase in lipid peroxidation and free radicals.
Dey D et al. [37]	pilot study	6 months	India	12 + 14	63.40 ± 8.44; 66.18 ± 8.29	no data available	2 g/day vitamin C supplementation	Exacerbation rates were found to be higher (OR: 5.26 [2.44–11.31], *p* < 0.0001) in standard therapy alone.

CRD: chronic respiratory disease; CRP: C-reactive protein; OR: odds ratio; TBARS: thiobarbituric acid-reactive substances; PC: protein carbonyl; IL: interleukin; TNF-α: tumor necrosis factor-α; SOD: superoxide dismutase; CAT: catalase; GSH: reduced glutathione; GPx: glutathione peroxidase.

**Table 2:** Studies on vitamin D supplementation in COPD.

Study	Design	Mean Follow-Up	Country	Sample Size	Average Age (Year)	Sex Male/Female	Intervention	Main Results
Rafiq R et al. [42]	double-blind, randomized, controlled trial	1 year	The Netherlands	155	67 ± 9; 65 ± 9	65.2%/34.8%	16,800 IU vitamin D3 once a week for 1 year	Vitamin D supplementation did not affect exacerbation rate: IRR: 0.90; 95% CI: 0.67–1.21.
Camargo CA Jr et al. [43]	randomized, double-blinded, placebo-controlled trial	3.3 years	New Zealand	5110	67	56%/44%	Monthly, high-dose vitamin D supplementation	Vitamin D supplementation had no effect on the exacerbation risk (HR: 1.08; 95% CI 0.84–1.39), but evidence of a probable benefit was found in those with severe vitamin D deficiency.
Alavi Foumani A et al. [44]	randomized, double-blinded clinical trial	4 months	Iran	63	67.9 ± 7.9; 68.4 ± 7.8	95.2%/4.8%	50,000 IU vitamin D3 once a week for 8 weeks and then once a month for 4 months	In the intervention group, a significant difference was observed in quality of life at 2 months (*p* < 0.001) and 6 months (*p* < 0.001).
Ghosh AJ et al. [45]	multicenter, longitudinal, observational study	5 years	United States	1544	57.07 ± 7.92; 62.09 ± 8.67	46.8%/53.2%	-	In adult smokers with and without COPD, vitamin D deficiency was associated with increased respiratory symptoms and poorer health-related quality of life at baseline, as well as increased frequency of exacerbations and airway wall thickening on chest CT scans.
Janssen R et al. [46]	double-blind, randomized, placebo-controlled trial	1 year	Belgium	142	68 ± 9; 68 ± 8	85.2%/14.8%	100,000 IU vitamin D3 supplementation every 4 weeks for 1 year	Vitamin D supplementation did not have a significant overall effect on elastin degradation compared to placebo.
Dastan F et al. [47]	randomized, double-blind, placebo-controlled trial	6 days	Iran	67	64.42 ± 7.58; 63.24 ± 8.41	85%/15%	300,000 IU of intramuscular vitamin D	IL-6 levels significantly decreased in the vitamin D vs. placebo group on the 6th day (*p* = 0.02); however, no significant differences were observed in IL-8 (*p* = 0.15) and hs-CRP (*p* = 0.24) levels, mMRC scale (*p* = 0.45) and mortality rates (*p* = 0.61).
Mølmen KS et al. [48]	double-blind, randomized clinical trial	28 weeks	Norway	95	68 ± 5	46.3%/53.7%	An initial 2 weeks with 10,000 IU/day, succeeded by 10 weeks with 2000 IU/day	Vitamin D3 supplementation did not affect muscular responses to resistance training in older adults with COPD.

IU: international unit; HR: hazard ratio; COPD: chronic obstructive pulmonary disease; CI: confidence interval; IRR: incidence rate ratio.

**Table 3:** Studies on antioxidant supplementation in COPD.

Study	Design	Mean Follow-Up	Country	Sample Size	Average Age (Year)	Sex Male/Female	Intervention	Main Results
De Benedetto F et al. [49]	double-blind, randomized, placebo-controlled clinical study	2 months	Italy	90	73 ± 7	76%/24%	Coenzyme Q10 (QTer^®^) and Creatine supplementation	Supplemented patients showed improvements in 6MWT (51 ± 69 versus 15 ± 91 m, *p* < 0.05), body cell mass and phase angle, sodium/potassium ratio, dyspnea indices and ADL score.
Beijers RJ et al. [50]	double-blind, randomized, placebo-controlled proof-of-concept study	4 weeks	The Netherlands	21	67 ± 9	57%/43%	Resveratrol supplementation (150 mg/day)	Plasma high-sensitivity C-reactive protein and kynurenine did not change after resveratrol supplementation. Muscle mitochondrial biogenesis regulators AMPK, SIRT1 and PGC-1α, mitochondrial respiration, Oxphos complexes, oxidative enzyme activities, and kynurenine aminotransferases were not improved by resveratrol.
Ghobadi H et al. [51]	randomized, double-blind, placebo-controlled clinical trial	12 weeks	Iran	46	62.04 ± 8.83	100% male	Crocin supplementation (30 mg/day)	COPD patients had decreased serum levels of TOS and NF-κB, and increased TAOC and 6MWD.
Gouzi F et al. [52]	randomized, double-blind, placebo-controlled clinical trial	28 days	France	57	62.4 ± 6.5	49%/51%	Antioxidant supplementation: α-tocopherol: 30 mg/day, ascorbate: 180 mg/day, zinc gluconate: 15 mg/day, selenomethionine: 50 μg/day	Supplementation increased the α-tocopherol/γ-tocopherol ratio and selenium, muscle strength (+11 ± 3%, *p* < 0.001), and serum total proteins (+7 ± 2%, *p* < 0.001), and it tended to increase the type I fiber proportion (+32 ± 17%, *p* = 0.07).
De Brandt J et al. [53]	double-blind, randomized, placebo-controlled trial	12 weeks	Belgium	40	65 ± 6	75%/25%	Beta-alanine supplementation	Beta-alanine supplementation increased muscle carnosine levels (+2.82 [1.49–4.14]; *p* < 0.001). However, accompanied beneficial changes in exercise capacity, quadriceps function, and muscle oxidative/carbonyl stress were not observed.
Lu MC et al. [54]	randomized, double-blind clinical trial	8 weeks	Taiwan	27	71 ± 2	-	Oligomeric proanthocyanidins extracted from grape seeds 150 mg/day suppl.	OPC supplementation significantly reduced the concentration of malondialdehyde, superoxide dismutase, and the total cholesterol/high-density lipoprotein cholesterol ratio.
Al-Azzawi MA et al. [40]	randomized, controlled, double-blind clinical trial	3 months	Egypt	91	55.18 ± 4.27	69%/31%	Treated with 1 g of 100% pure cold-pressed black seed oil, orally, twice daily, in addition to standard COPD medication	Significant reduction in oxidant and inflammatory markers: TBARS, PC content, IL-6, TNF-α. A significant increase in antioxidants SOD, CAT, GSH, GPx vitamin C, and E, and a significant improvement in PFTs versus control group and baseline levels.
Han MK et al. [55]	randomized clinical trial	1 week	Michigan	9	68 ± 6	56%/64%	Quercetin at 500, 1000 or 2000 mg/day	Quercetin was safely tolerated up to 2000 mg/day based on lung function tests, blood profiles, and COPD assessment test questionnaires.
Aslani MR et al. [56]	randomized, double-blind, placebo-controlled trial	12 weeks	Iran	57	61 ± 8	100% male	Crocin supplementation 15 mg twice daily	Crocin improved PFT (*p* < 0.05) and 6MWD (*p* < 0.001) and increased serum levels of IL-6 and TNF-alfa.

6MWT: 6 min walking distance; AMPK: adenosine monophosphate-activated protein kinase; SIRT1: Sirtuin 1; PGC-1α: peroxisome proliferator-activated receptor-gamma coactivator (PGC)-1 alpha; COPD: chronic obstructive pulmonary disease; TOS: total oxidant status; NF-κB: nuclear factor kappa B; TAOC: total antioxidant capacity; OPC: oligomeric proanthocyanidin; TBARS: thiobarbituric acid-reactive substances; PC: protein carbonyl; IL-6: interleukin-6; TNF-α: tumor necrosis factor-α; SOD: superoxide dismutase; CAT: catalase; GSH: glutathione; GPx: glutathione peroxidase; PFT: pulmonary function test.

**Table 4:** Studies on omega−3 polyunsaturated fatty acid supplementation in COPD.

Study	Design	Mean Follow-Up	Country	Sample Size	Average Age (Year)	Sex Male/Female	Intervention	Main Results
Kim JS et al. [58]	prospective, randomized, placebo-controlled, double-blinded superiority trial	6 months	Columbia	40	67.5 (6.5)	55%/45%	Daily administration of high-dose fish oil capsules for 6 months	Randomization of n-3 PUFAs for 6 months did not change systemic endothelial function in COPD. More participants in the fish oil arm reported at least a 4 point improvement in the SGRQ.
Engelen M et al. [59]	randomized, double-blind, placebo-controlled 3-group design study trial	4 weeks	USA	32	-	-	High dose (3.5 g) of EPA + DHA, a low dose (2.0 g) of EPA + DHA, or placebo via gel capsules	Daily *n*-3 PUFA supplementation induces a shift toward a positive daily protein homeostasis in patients with COPD in a partially dose-dependent way. Extremity lean mass increased, but muscle function did not.
Ogasawara T et al. [60]	prospective, randomized, controlled trial	12.6 ± 4.9 days	Japan	45	77.4 ± 9.7	91%/9%	Oral administration of 1 g/day of EPA-enriched nutrition supplementation	Insignificant increase in LBMI and SMI in the EPA group compared with the control group. The change in the SMI was significantly correlated with the length of hospitalization in the EPA group.

COPD: chronic obstructive pulmonary disease; EPA: eicosapentaenoic acid; DHA: docosahexaenoic; PUFA: polyunsaturated fatty acid; SGRQ: St George’s Respiratory Questionnaire; LBMI: lean body mass index; SMI: skeletal muscle mass index; USA: United States of America.

**Table 5:** Studies on magnesium and sodium nitrate supplementation in COPD.

Study	Design	Mean Follow-Up	Country	Sample Size	Average Age (Year)	Sex Male/Female	Intervention	Main Results
Zanforlini BM et al. [61]	double-blind, randomized, controlled clinical study	6 months	Italy	49	72.6 ± 9.9	77.6%/22.4%	300 mg/day magnesium citrate	Oral magnesium supplementation may have a potential anti-inflammatory role, CRP (β = −3.2, 95% CI −6.0, −0.4, *p* = 0.03).
Beijers RJHCG et al. [62]	double-blind, randomized, cross-over, placebo-controlled trial	7 days	The Netherlands	20	66.6 ± 7.5	72.2%/27.8%	Sodium nitrate (8 mmol/day)	Acute as well as 7-day sodium nitrate supplementation does not alter mechanical efficiency, blood pressure or cardiac biomarkers in mild-to-moderate COPD patients.

CRP: C-reactive protein; COPD: chronic obstructive pulmonary disease; CI: confidence interval.

**Table 6:** Exogenous and endogenous sources of reactive oxygen species.

Exogenous Agents	Reactive Oxygen Species	Endogenous Agents
Cigarette smoke	Superoxide anion	Inflammation
Pollutants	Hydrogen peroxide	Organelle damage
Radiation	Hydroxyl radical	Mitochondria
Drugs	Hypochlorous acid	Cell metabolism
Foods	Hydroxide ion	Peroxisomes

“Adapted with permission from Ref. [65]. Copyright 2018, copyright Boukhenouna S, et al.”

## Data Availability

Data sharing is not applicable to this article as no new data were created or analyzed in this study.

## Abbreviations

25(OH)D: 25-hydroxyvitamin D; 6MWT: 6 min walking distance; aHR: adjusted hazard ratio; AMPK: adenosine monophosphate-activated protein kinase; BMI: body mass index; Ca: calcium; CAT: catalase; CAT: COPD quality of life questionnaire; CI: confidence interval; COPD: chronic obstructive pulmonary disease; CoQ10: Coenzyme Q10; CRP: C-reactive protein; DALYs: disability-adjusted life years; DHA: docosahexaenoic; EMP: endothelial microparticle; EPA: eicosapentaenoic acid; FEV_1_: forced expiratory volume in one second; FFM: fat-free mass; GPx: glutathione peroxidase; GSH: glutathione; GSH: reduced glutathione; HDL-C: high-density lipoprotein cholesterol; HR: hazard ratio; ICS: inhaled corticosteroids; IL-6: interleukin-6; IRR: incidence rate ratio; IU: international unit; LABA: long-acting beta2-agonists; LBM: lean body mass; LBMI: lean body mass index; MMP9: matrix metalloproteinase-9; NF-κB: nuclear factor kappa B; NHANES: National Health and Nutrition Examination Survey; NT-proBNP: *N*-terminal prohormone of brain natriuretic peptide; OPC: oligomeric proanthocyanidin; OR: odds ratio; PC: protein carbonyl; PFT: pulmonary function test; PGC-1α: peroxisome proliferator-activated receptor-gamma coactivator; PUFA: polyunsaturated fatty acid; RCT: randomized controlled trials; ROS: reactive oxygen species; SGRQ: St George’s Respiratory Questionnaire; SIRT1: Sirtuin 1; SMI: skeletal muscle mass index; SOD: superoxide dismutase; TAOC: total antioxidant capacity; TBARS: thiobarbituric acid-reactive substances; TC: total cholesterol; TNF-α: tumor necrosis factor-α; TOS: total oxidant status; USA: United States of America; WHO: World Health Organization.
